# Americans on the Voluntary System: The Criticisms of Visiting Surgeons

**Published:** 1914-08-08

**Authors:** 


### AMERICANS ON THE VOLUNTARY SYSTEM.

### The Criticisms of Visiting Surgeons.

### A PLEA FOR THE PAY-WARD.

Dr. Franklin H. Martin, the general secretary of the Clinical Congress of Surgeons of North America, gave some remarkable criticisms of our voluntary hospitals to a representative of the *Times*, which we reproduce here from that journal. It is true that they largely resolve themselves into a plea for a graduated system of pay-wards for all classes of patients, which obtains in America, but from reading the actual remarks institutional officers in this country will be enabled to judge how our system and our hospitals present themselves to American eyes.

#### Exclusion of Middle-class Patients.

"The condition that amazes the surgeon of North America," Dr. Franklin Martini is reported to have said, "is the method of conduct of hospitals in London. A group of these great palatial buildings, occupying acres of the most valuable land in the capital of the world, are devoted exclusively to the care of the non-paying patient, and exclude from the advantages of these equipments the patient of moderate means, and render them prohibitive to patients of wealth. These hospitals with their large endowments should furnish the most elaborate equipment of operating-room and laboratory diagnostic facilities that can be produced by an institution with unlimited resources. With this equipment the leading surgeons of London spend a third of their time and energy in caring for individuals presumably unable to pay for their hospital accommodation or for the surgery that is performed upon them.

#### Equipment of Private Hospitals.

"This same surgeon, who of necessity is in great demand by the paying public, is obliged to operate upon patients of means in private hospitals that cannot afford the equipment of the endowed hospital, or in the homes of their patients with imperfect, makeshift arrangements. Why should a great group of the less fortunate class of the people be forced to accept free beds and pauperism, when they could well afford, and in many cases be glad to pay, a small sum that would cover their hospital fees? Why should the next higher class of individuals be deprived of hospital facilities of any kind between the high-priced nursing home and the pauper bed? Why should the wealthy patient be unable to take advantage of a highly-developed operating-room technique?

#### Penalising the Surgeons.

"Such a system, we think, inflicts an almost prohibitive penalty upon the great surgeons of the great hospitals, as it necessitates their operating not only upon the poor who are operated upon in their well-equipped hospitals, but also upon the middle-class in the poorly-equipped nursing home, and a third-class in the wealthy homes or the better-equipped nursing home. Each of these surgeons is obliged to operate in three distinct environments every day of his life, with different assistants, with different operating rooms, and in many cases no operating rooms at all.

#### The American Contrast.

"On the Continent of America the best hospitals are equipped to care for all classes of patients. The operating rooms are perfectly equipped, and can be utilised for pauper and multi-millionaire alike. Women in Chicago whose wealth can be counted by millions, go to the hospital to be confined, because they know that at the hospital the surgeon works in his accustomed environment and he is completely equipped for his work. A week later the patient returns to her home on an ambulance accompanied by her nurse. In the hospitals of the United States surgeons, whether on the staff or not, are encouraged to bring their private patients for operation; and the result is that we have ten surgeons for one in England.

#### Wealthy Patients in American Hospitals.

"Sometimes an American surgeon will operate on three wealthy patients in one morning, before the students in the common operating theatre, perhaps receiving 200 guineas for each operation. These wealthy patients are removed to palatial rooms in the hospital. Between these operations the poor are brought in and operated upon with the same skill and care as that given to the wealthy. When President Roosevelt was shot he was taken to a general hospital, and we knew from the first that he was all right.

#### A Pay-Ward System Predicted.

"In this country the non-paying patient occupying the large hospitals is in a position to be better cared for than the middle class or the wealthy class. Naturally it would be difficult for the English public to understand how a hospital could be so equipped that all classes could be accommodated in it with definite comfort to each. I am quite sure that any English surgeon, however, would admit that he could do better work in one well-regulated operating room than in several, or in private homes. I predict that the English middle class and the wealthy class will soon demand the same high-class hospital accommodations that are accorded exclusively to the poor."

The above criticisms are well worth reflecting on, for they are not merely constructive in the sense of presenting a picture of the advantages of the graduated pay-ward system to patients and surgeons alike, but they point out also one aspect which is less appreciated, namely, that by bringing the wealthy patient into personal contact with the hospital, valuable gifts and life-long support are often received, and legacies also attracted.

